# Unveiling Hidden Genetic Architectures: Molecular Diagnostic Yield of Whole Exome Sequencing in 50 Children With Autism Spectrum Disorder Negative for Copy Number Variations

**DOI:** 10.1155/genr/5724454

**Published:** 2025-07-03

**Authors:** Zhiwei Wang, Yali Zhao, Shuting Yang, Yongan Wang, Leilei Wang

**Affiliations:** Department of Prenatal Diagnosis, Lianyungang Maternal and Child Health Hospital, Lianyungang, Jiangsu 222000, China

**Keywords:** autism spectrum disorders, genetic etiologies, whole exome sequencing

## Abstract

Autism spectrum disorders (ASDs) are heterogeneous neurodevelopmental conditions with complex genetic etiologies. Recent advances in whole exome sequencing (WES) have enabled comprehensive detection of clinically relevant variants, particularly single-nucleotide variations (SNVs) and InDels, in ASD genetic diagnostics. Here, we performed WES on 50 Chinese children with ASD who tested negative for copy number variants (CNVs). The analysis achieved a diagnostic yield of 10% (5/50 cases). All SNVs and InDels were loss-of-function (LOF) and were slightly more frequent among females (male vs. female: 9.3% vs. 14.3%). A total of five causative genes (*PRODH9, PTEN, DEPDC5, SATB2,* and *CYFIP1*) were identified in this study. Variants in ASD-associated genes (*CHD8, FOXP1,* and *SHANK1*) and genes linked to other neurodevelopmental disorders (*CDH15, GATAD2B,* and *SHROOM4*) were also detected. Despite the small sample size, our findings contribute partially to the dataset on the phenotype and genetic etiology of ASD and underscore WES as a critical tool for elucidating genetic etiologies in CNV-negative ASD cohorts.

## 1. Introduction

Autism spectrum disorder (ASD) represents a group of neurodevelopmental conditions with impairments in reciprocal social interaction, communication, and restricted/repetitive behaviors in early childhood. ASD affects approximately 1% of the global population, with a marked male predominance (4:1) [1, 2]. Its clinical heterogeneity and multifactorial etiology, which encompass genetic, epigenetic, and environmental factors, pose significant diagnostic challenges [3, 4]. It is believed that genetic factors play a key role in the pathogenesis of ASD ranging from single-nucleotide variants (SNVs), copy number variations (CNVs), to large chromosome imbalances [5, 6]. Delineating the genetic architecture of ASD is a substantial challenge due to its genetic polymorphism and phenotypic heterogeneity.

Over the past 15 years, significant advances have been made in understanding CNVs in individuals with ASD. In recent years, chromosomal microarray (CMA) techniques, including Array-CGH and SNP-array, have emerged as robust, high-resolution methods of analysis, accounting for approximately 5%–10% of ASD cases [7–9]. To date, over 40 recurrent CNVs have been consistently associated with ASD [10]. Notably, abnormalities have been identified at multiple loci on chromosomes 16p11.2, 15q11-q13, 17p11.2, and 22q11-q13, frequently involving genes such as *PTCHD1, NRXN1, NLGN3, SHANK3,* and *SHANK1* [11–13]. However, it is difficult to detect short CNVs smaller than 500 bp or SNVs, and a causal relationship is often hard to prove between the detected genetic variation and ASD.

In the last decade, the advent of next-generation sequencing (NGS) and whole exome sequencing (WES) has opened new avenues in autism research. WES accelerates diagnosis by simultaneously sequencing all exons or coding regions of the genome, making it a powerful tool for identifying rare SNVs and insertions/deletions (InDels) that may illuminate the complex genetic architecture of autism [14]. Recent studies have highlighted the significant role of de novo SNVs in increasing the risk of ASD. Notably, many de novo SNVs have been identified in individuals with sporadic ASD, affecting genes such as *CHD8, DYRK1A, ANK2, NTNG1,* and *GRIN2B* [15, 16]. Research involving WES in parent–child trios has demonstrated that de novo mutations contribute substantially to molecular diagnoses, with a predominant occurrence of loss-of-function (LOF) variants [17–19]. WES has proven to complement CMA detection in identifying candidate genes and enhancing our understanding of the genetic landscape of ASD. A comparative study of molecular diagnostics using CMA and WES in children with ASD revealed that the diagnostic yields of both methods were comparable among heterogeneous samples [20]. However, the genetic landscape of CNV-negative ASD cohorts remains underexplored by conventional methods.

This study aimed to assess the diagnostic yield of WES in 50 sporadic CNV-negative ASD children. We hope to expand the genetic spectrum of ASD by identifying novel SNVs and InDels in ASD-associated genes.

## 2. Materials and Methods

### 2.1. Patients

Children with ASD were recruited from Lianyungang Maternal and Child Health Hospital. All participants were diagnosed based on the DSM-IV criteria. The diagnostic evaluation included a comprehensive clinical assessment utilizing the Autism Diagnostic Interview, Revised (ADI-R), which is recognized as the gold standard for ASD diagnosis [21, 22]. These children underwent CMA using the Affymetrix CytoScan 750K Array (USA). Individuals without large CNVs (CNVs > 200 kb for deletion and > 500 kb for duplication) were included in the cohort. Ultimately, 50 unrelated children with ASD (43 males and 7 females; aged 2–9 years) were enrolled. The study protocol was approved by the Ethics Committee of Lianyungang Maternal and Child Health Care Hospital (Approval no: LW2021016), and written informed consent was obtained from all parents or guardians.

### 2.2. DNA Sample and WES

Genomic DNA was extracted from peripheral blood using QIAamp DNA Blood Mini kit (QIAGEN, Germany) according to the manufacturer's protocol. Then, the isolated gDNA samples were randomly fragmented by a Covaris sonicator. They were end-repaired, enriched, and circularized into DNA nanoballs. Subsequently, WES was performed using the BGISEQ-500 platform (BGI, Shenzhen, China). Raw image files were processed to produce pair-end reads for each individual using the default parameters of the base calling software developed for BGISEQ-500.

### 2.3. Read Mapping and Variant Analysis

After data filtering, the clean data of each sample was mapped to the human reference genome (GRCh37/HG19) using a Burrows–Wheeler Aligner (BWA V0.7.15) tool. Picard tools (V2.5.0, https://broadinstitute.github.io/picard/) were used to remove duplicate reads. All genomic variations, including single-nucleotide polymorphisms (SNPs) and InDels, were detected and filtered by GATK Haplotype Caller (V4.2.6.1) (Broad Institute, Cambridge, MA, USA). Given our cohort size (*n* = 50 sporadic cases) falls below the minimum requirement for reliable VQSR application (*n* ≥ 100 recommended by GATK Best Practices), we implemented a stringent hard filtering approach: quality thresholds including QUAL ≥ 30, QD ≥ 2.0, FS ≤ 60 (SNVs) or ≤ 200 (InDels), MQ ≥ 40, and ReadPosRankSum ≥ −8.0; and coverage thresholds including DP ≥ 10× (per-sample) and GQ ≥ 20. Subsequently, variants were annotated using SnpEff 5.0 with the RefSeq annotations on GRCh37 [23]. Potential disease-causing mutations were predicted using the sorting intolerant from tolerant (SIFT) algorithm. Data were filtered with several variant databases, including dbSNP (https://www.ncbi.nlm.nih.gov/projects/SNP/), the 1000 Genomes Project (https://ftp-trace.ncbi.nih.gov/1000genomes/ftp/release), the NHLBI-ESP6500 database (https://evs.gs.washington.edu/EVS/), the gnomAD (https://gnomad-old.broadinstitute.org/), and the Exome Aggregation Consortium (ExAC) (https://exac.broadinstitute.org/). Candidate mutations were expected to be absent from these databases. The conservation analysis of amino acid sequences was aligned using ClustalW2 (https://www.ebi.ac.uk/Tools/msa/clustalw2/). Moreover, candidate variants were detected in all enrolled family members via Sanger sequencing (data not shown).

### 2.4. Sanger Sequencing

Pathogenic variants in candidate genes were confirmed by Sanger sequencing. DNA samples were sequenced using the PE Big Dye Terminator Cycle Sequencing Kit on an ABI Prism 3500 analyzer (Applied Biosystems). The data were analyzed using the Sequencher Version 4.9 software.

## 3. Results

### 3.1. Demographics and Diagnostic Yield

The characteristics of 50 children diagnosed as ASD are summarized in Table 1. First, we analyzed the CNVs identified through WES. All of the 27 CNVs were classified as benign or of uncertain clinical significance (Supporting Information, Table S1). The conclusive diagnoses in our cohort were based on pathogenic or likely pathogenic gene variants. Among ASD patients, 10 children were under 3 years old, 34 were between 3 and 6 years old (diagnostic rate = 11.8%), and 6 were older than 6 years (diagnostic rate = 16.7%). Females exhibited a higher yield (14.3% vs. 9.3% in males) despite the male-biased cohort ratio of 43:7. We confirmed six pathogenic or likely pathogenic variants across five genes: *PRODH9* (*c.1292G > A*, *c.1322T* > *C*), *PTEN* (*c.487dupA*), *DEPDC5* (*c.3092C* > *A*), *SATB2* (*c.1166G > A*), and *CYFIP1* (*c.3401_3414 del*) (see Table 2). All were LOF with autosomal dominant (80%) or recessive (20%) inheritance. It is worth noting that *PTEN* and *DEPDC5* were found to be mutated in multiple cases within our ASD cohort.

### 3.2. Variant Spectrum and Genes Related to ASD

WES uncovered a heterogeneous mutational landscape within the ASD cohort, identifying 77 SNVs, 2 insertions, and 6 short deletions across 40 identified associated genes (Table 3). 25 ASD-risk genes contained variants of uncertain clinical significance (VUS), suggesting a potential phenotypic overlap between ASD and broader NDD mechanisms (Table 4). In order to better validate these classifications, we systematically categorized all ASD-risk genes referenced in our study using the established SFARI Gene Database (https://gene.sfari.org/database/human-gene/). Specifically, genes were classified based on the SFARI Gene Score categories as follows: Score 1 (*high confidence*), Score 2 (*strong candidate*), and Score S (*syndromic*). Among the 25 genes associated with ASD, 7 genes were found to be highly confident (Score 1), 2 were strong candidate genes (Score 2), 14 genes scored 1, S and 2 genes were associated with the syndrome (Score S).

Moreover, the identified variants were enriched in biological pathways crucial for neurodevelopment. Key genes such as *SHANK1, FOXP1, PTEN, ZSWIM6, CIC, SYNGAP1, NRXN3, DEPDC5, MYT1L, EHMT1, GRM7,* and *MED13L* are associated with dendritic morphogenesis and synaptic signaling. In contrast, *CHD8, CHD2, CHD7*, and *POGZ* are implicated in chromatin remodeling. Furthermore, ADNP, and DEAF1 function as transcription factors involved in nervous system development, while *NAA15, ASH1L, SETD5, SETD2*, and *KMT2C* are linked to networks governing posttranslational modifications [24, 28, 31].

### 3.3. Genetic Variants in Neurological Disorder–Associated Genes

Beyond the core ASD-associated genes identified in this study, our analysis revealed a distinct subset of rare variants occurring in loci primarily associated with other neurodevelopmental conditions (Table 5). Notably, these variants were identified in genes including *CDH15* (mental retardation)*, GATAD2B* (mental retardation), *SEMA3E* (CHARGE syndrome)*, PDE4D* (Acrodysostosis 2, with or without hormone resistance)*, SHROOM4* (Stocco dos Santos X-linked mental retardation syndrome)*, DLG3* (mental retardation), and *ERE* (neurodevelopmental disorder), all of which were believed to be potentially novel ASD candidate genes but lack previous association with ASD in major genomic databases. Several genes were also found to be rarely associated with autism in literature databases such as FRMPD4. The *FRMPD4* mutations are usually associated with a substantial degree of increased risk and consistently linked to additional characteristics not required for an ASD diagnosis. This is consistent with the clinical symptoms of the cases in this study having other co-occurring neurodevelopmental features.

## 4. Discussion

### 4.1. Diagnostic Yield and Sex Disparity

NGS, as a new approach, was being used from 2007. WES seemed to be frequently used to identify rare SNVs contributing to the risk of multiple disorders including ASD. Undoubtedly, identifying novel SNVs and InDels could make up for the deficiency of CMA in expanding the genetic spectrum of ASD. In our current study, WES was further implemented among 50 children diagnosed as ASD with negative findings of CMA. All variants with a predicted damaging effect were validated by Sanger sequencing. Genetic etiology was identified in five of 50 children with an overall detection rate of 10%, which was similar to the results observed among sporadic ASD (8.4%), as well as those focusing on either de novo or inherited variations, ranging from 6.3% to 13.8% [15, 20, 32]. A detection rate was reflected in the 3–6-year-old children (11.8%). Du et al. reported a detection rate of 16.7% in 3–6-year-old children with ASD [17]. According to the Centers for Disease Control and Prevention (CDC), the average age of diagnosis is 4.5 years, when symptoms in the three core domains become apparent (CDC, 2009). An unusual finding emerging from this study showed that the diagnostic rates of male and female were 9.3% and 14.3% in spite of a predominant male to female ratio (about 4:1) according to previous studies [6]. One possible explanation was that limited data did not reflect the real situation. Larger cohorts are needed to validate this trend.

### 4.2. Gene-Phenotype Correlations

All of the identified SNVs and InDels were LOF variations. Five genes (*PRODH, PTEN, DEPDC5, SATB2,* and *CYFIP1*) were identified among patients diagnosed as ASD. All genes with presumed causative mutations identified here were previously reported in ASD. *PRODH* may be involved in 22q11-associated psychiatric and behavioral phenotypes. Several reports suggested that a homozygous mutation in the *PRODH* gene could cause hyperprolinemia, Type I disease, which usually showed with neurologic manifestations, including ASD and seizures. *PRODH* was also associated with psychiatric disorders such as schizophrenia when there is a heterozygous mutation [33]. We identified two variants (c.1292G > A and c.1322T > C) in the *PRODH* gene of a 7-year-old girl with ASD. The variants were believed to be the cause of ASD. There is evidence that macrocephaly/autism syndrome (# 605309) is caused by a heterozygous mutation in the *PTEN* gene (601,728) on chromosome 10q23. O'Roak et al. identified 3 de novo mutations in the *PTEN* gene while sequencing 44 candidate genes among 2446 ASD probands, and there were 2 missense and 1 frameshift mutation identified [34]. In a prospective study of Turkish children with ASD and macrocephaly conducted by Kaymakcalan et al., the prevalence of *PTEN* mutations was found to be 3.8% (including variants of uncertain significance) or 2.29% (excluding variants of uncertain significance) [35]. Heterozygous mutations in the *DEPDC5* gene typically give rise to familial focal epilepsy (# 604364). Burger et al. reported the first case to link ASD to a mutation of *DEPDC5* [36]. Numerous mutations, including CNVs, SNVs, and chromosomal rearrangements that disrupt *SATB2* have been reported in ASD cases, which provided strong evidence that loss of *SATB2* function could contribute to ASD [37]. The *CYFIP1* gene is located in a chromosomal region linked to various neurological disorders, including intellectual disability, autism, and schizophrenia [38]. Noroozi et al. compared expression levels of CYFIP1 in ASD patients and healthy subjects, which further supports for contribution of CYFIP1 in the pathogenesis of ASD and potentiates it as a peripheral marker for ASD diagnosis [39]. Through the analysis above, the genes associated with autism are also related to other psychiatric conditions, suggesting that there is a common genetic background for psychiatric disorders.

Although only a few causative ASD genes have been detected, substantial genes related to ASD were identified (Table 4). According to the SFARI Gene Scoring system, 7 genes with SFARI Score 1 (e.g., *SETD2, ASH1L,* and *NRXN3*) demonstrate strong evidence for direct causality in core ASD phenotypes. 14 genes with SFARI Score 1, S (for example) are characterized by defined neurodevelopmental syndromes with ASD as a comorbidity. Genes such as *ZSWIM6* and *DEPDC5* that scored S-category indicated playing roles in motor-neural circuits beyond core ASD. SFARI Category 2 Genes (*SHANK1* and *GRM7*) showed reduced phenotypic specificity compared to Category 1 genes. SFARI scores stratify ASD-risk genes by phenotypic breadth: Score 1 genes drive core ASD/ID, while 1, S/S genes dictate syndromic disorders with ASD comorbidity. This delineation refines prognosis, surveillance, and therapeutic strategies. Future studies should integrate functional assays to reclassify VUS in high-scoring genes, enhancing clinical utility.

The identified variants were enriched in biological pathways critical for neurodevelopment. Genes implicated in dendritic morphogenesis and synaptic signaling, such as *SHANK1, FOXP1, PTEN,* and *ZSWIM6* were considered as potential novel candidates for ASD [24, 28, 31]. A heterozygous de novo *FOXP1* variant was identifed with exome sequencing in a patient with autism, intellectual disability, and severe speech and language impairment [24]. In addition, *CHD8, CHD2, CHD7,* and *POGZ* were also identified to regulate chromatin remodeling [24, 28]. *CHD8* is a chromatin regulator enzyme that is essential during fetal development. Allelic variants of *CHD8* are associated with ASD [24]. *ADNP* and *DEAF1* were involved in nervous system development as transcription factors. ADNP is required for neural induction and differentiation by enhancing Wnt signaling [24]. In addition, *NAA15, ASH1L, SETD5, SETD2*, and *KMT2C* are linked to networks governing posttranslational modifications. *SETD5* and *SETD2* genes share functional similarities such as playing multiple roles in the central nervous system through histone and nonhistone methylation, and their dysfunction leads to complex neurodevelopmental disorders [28, 31]. This provides additional evidence that the investigated ASD-related genes may be candidate genes for ASD.

### 4.3. Potential Autism-Related Genes

Additional genes identified through WES have been previously linked to neuronal functions but lack previous association with ASD in major genomic databases including CDH15, GATAD2B, SEMA3E, PDE4D, SHROOM4, DLG3, and ERE. These genes have mainly been reported to be associated with other nervous system development. A study by Shieh et al. involving 50 patients with neurodevelopmental disorders identified several genetic variants in *GATAD2B* as key contributors to the diverse clinical phenotypes of these disorders [40]. Disruptions in the newly identified *SHROOM4* gene are associated with X-linked mental retardation [41]. DLG3 encodes the synapse-associated protein 102 (SAP102), a member of the membrane-associated guanylate kinase (MAGUK) protein family, and is implicated in dystonia-Parkinsonism syndromes [42]. Several genes were also found to be rarely associated with autism in literature databases such as FRMPD4. The FRMPD4 mutations are usually associated with a substantial degree of increased risk and consistently linked to additional characteristics not required for an ASD diagnosis. This is consistent with the clinical symptoms of the cases in this study having other co-occurring neurodevelopmental features. Currently, data regarding the sensitivity and specificity of these genes appear insufficient, necessitating further validation of their genotype–phenotype relationships.

## 5. Conclusion

WES enhances genetic diagnosis in CNV-negative ASD, uncovering both known and novel pathogenic variants. This study presents a comprehensive exome analysis of 50 children diagnosed with sporadic ASD. Despite the small sample size, our findings contribute partially to the dataset on the phenotype and genetic etiology of ASD and underscore WES as a critical tool for elucidating genetic etiologies in CNV-negative ASD cohorts. However, there still exists a knowledge gap regarding the diagnostic yield of WES specifically for autistic features. Therefore, it is necessary to establish causal relationships between candidate genes and ASD patients.

## Figures and Tables

**Table 1:** Diagnostic rate of children with different characteristics.

Characteristic	Children with ASD	
	No.	Diagnostic rate
Age at testing		
< 3	10	0/10 (0)
3–6	34	4/34 (11.8%)
≥ 6	6	1/6 (16.7%)
Sex		
Male	43	4/43 (9.3%)
Female	7	1/7 (14.3%)

**Table 2:** Molecular findings in pediatric patients exhibiting pathogenic/likely pathogenic results.

Case no.	Sex	Age at testing	Phenotypic feature	Gene	Sequence variant	Amino acid variant	Hom/het	Interpretation	ACMG classification	Molecular diagnosis
1	Female	7y	ASD	*PRODH9* (606,810, AR)	c.1292G > A	p.R431H	Het	Nonsynonymous SNV	Likely pathogenic	Hyperprolinemia, Type I
					c.1322T > C	p.L441P	Het	Nonsynonymous SNV		
2	Male	5y	ASD	*PTEN* (601,728, AD)	c.487dupA	p.D162 fs	Het	Frameshift insertion	Pathogenic	Macrocephaly/autism syndrome
3	Male	5y	ASD	*DEPDC5* (614,191, AD)	c.3092C > A	p.P1031H	Het	Nonsynonymous SNV	Likely pathogenic	Epilepsy, familial focal, with variable foci 1
4	Male	3y	ASD	*SATB2* (608,148, AD)	c.1166G > A	p.R389H	Het	Nonsynonymous SNV	Likely pathogenic	Glass syndrome
5	Male	3y	ASD	*CYFIP1* (606,322, AD)	c.3401_3414 del	p.M1134 fs	Het	Frameshift deletion	Likely pathogenic	Autism

**Table 3:** Summary of the number of variants detected in this study.

Mutations	SNV		Insertion		Deletion	
	Synonymous SNV	Nonsynonymous SNV	Frameshift insertion	Nonframeshift insertion	Frameshift deletion	Nonframeshift deletion
No.	2	75	1	1	2	4

**Table 4:** List of genes related to ASD.

Gene	Sequence variant	Amino acid variant	Interpretation	Hom/het	ACMG classification	Molecular diagnosis	SFARI gene score	Reported link to ASD	
								Mutation type	References
CHD8	c.5666G > A	p.R1889H	Nonsynonymous SNV	Het	VUS	Susceptibility to autism	1, S	Frameshiſt InDel, missense	[24]
	c.2318G > A	p.R773Q	Nonsynonymous SNV	Het	VUS	Susceptibility to autism			
	c.6769G > A	p.D2257N	Nonsynonymous SNV	Het	VUS	Susceptibility to autism			

PTEN	c.493T > G	p.F165V	Nonsynonymous SNV	Het	VUS	Macrocephaly/Autism syndrome	1, S	Splice site	[24]

FOXP1	c.125C > T	p.P42L	Nonsynonymous SNV	Het	VUS	Mental retardation with language impairment and with or without autistic features	1, S	Frameshift	[24, 25]
	c.1696G > A	p.A566T		Het	VUS	Mental retardation with language impairment and with or without autistic features			

SHANK1	c.1619C > T	p.S540F	Nonsynonymous SNV	Het	VUS	Autism spectrum disorder	2	Microdeletion	[24]
	c.1721G > A	p.R574H		Het	VUS	Autism spectrum disorder			

ZSWIM6	c.3035C > T	p.T1012M	Nonsynonymous SNV	Het	VUS	Neurodevelopmental disorder with movement abnormalities, abnormal gait, and autistic features	S	Nonsense	[26]

CIC	c.496C > T	p.P166S	Nonsynonymous SNV	Het	VUS	Mental retardation	1	Missense	[27]
	c.1526C > A	p.P509Q		Het	VUS	Mental retardation			

CHD2	c.875C > T	p.P292L	Nonsynonymous SNV	Het	VUS	Epileptic encephalopathy, childhood-onset	1, S	Frameshiſt	[28]

MED13L	c.4897G > A	p.G1633S	Nonsynonymous SNV	Het	VUS	Mental retardation and distinctive facial features with or without cardiac defects	1, S	Frameshiſt	[24]

ADNP	c.3205A > G	p.I1069V	Nonsynonymous SNV	Het	VUS	Helsmoortel–Van der Aa syndrome	1, S	Frameshift or nonsense	[24]
	c.2782G > C	p.D928H	Nonsynonymous SNV	Het	VUS	Helsmoortel–Van der Aa syndrome			
	c.2653_2655del	p.885_885del	Nonframeshift deletion	Het	VUS	Helsmoortel–Van der Aa syndrome			

SETD2	c.557C > T	p.P186L	Nonsynonymous SNV	Het	VUS	Luscan–Lumish syndrome	1	Frameshift deletion	[28]
	c.2283G > A	p.M761I		Het	VUS	Luscan–Lumish syndrome			

AFF2	c.1423C > T	p.P475S	Nonsynonymous SNV	Het	VUS	Mental retardation, X-linked recessive	1	Microdeletion	[24]
	c.493A > G	p.N165D		Het	VUS	Mental retardation			

SYNGAP1	c.2275A > G	p.M759V	Nonsynonymous SNV	Het	VUS	Mental retardation	1, S	Missense mutations, splicing mutations	[28]
	c.1410G > A	p.M470I		Het	VUS	Mental retardation			

NRXN3	c.1721G > A	p.R574H	Nonsynonymous SNV	Het	VUS	Autism spectrum disorder	1	Microdeletion	[24]

DEAF1	c.1526G > A	p.R509Q	Nonsynonymous SNV	Het	VUS	Mental retardation	1, S	Splice acceptor mutation	[24]

POGZ	c.412G > A	p.V138M	Nonsynonymous SNV	Het	VUS	White–Sutton syndrome	1, S	Missense variants	[24]

NAA15	c.2101A > G	p.I701V	Nonsynonymous SNV	Het	VUS	Mental retardation	1, S	Del/ins mutation	[29]

ASH1L	c.2795G > A	p.S932N	Nonsynonymous SNV	Het	VUS	Mental retardation	1	SNV	[28]

SETD5	c.4181C > G	p.A1394G	Nonsynonymous SNV	Het	VUS	Mental retardation	1, S	SNV, duplication and deletion	[28]
	c.3338A > G	p.H1113R		Het	VUS	Mental retardation			

DEPDC5	c.1612C > T	p.P538S	Nonsynonymous SNV	Het	VUS	Epilepsy	S	SNV	[30]

MYT1L	c.483_488del	p.161_163del	Nonframeshift deletion	Het	VUS	Mental retardation	1	Duplication	[28]
	c.1809C > T	p.L603L	Synonymous SNV	Het	VUS	Mental retardation			

KMT2C	c.351_352insTCGGCA	p.N118delinsSAN	Nonframeshift insertion	Het	VUS	Kleefstra syndrome 2	1, S	SNV, del	[24]
	c.11056A > G	p.N3686D	Nonsynonymous SNV	Het	VUS	Kleefstra syndrome 2			

EHMT1	c.980A > C	p.K327T	Nonsynonymous SNV	Het	VUS	Kleefstra syndrome	1, S	SNV	[31]

GRM7	c.2420delT	p.I807fs	Frameshift deletion	Het	VUS	Attention deficit–hyperactivity disorder	2	Deletion	[24]

ATRX	c.1322_1324del	p.441_442del	Nonframeshift deletion	Het	VUS	Alpha-thalassemia/mental retardation syndrome, X-linked	1	SNV	[30]

CHD7	c.7145C > T	p.T2382M	Nonsynonymous SNV	Het	VUS	CHARGE syndrome	1, S	Missense variants	[31]
	c.7170T > G	p.D2390E		Het	VUS	CHARGE syndrome			
	c.3979A > G	p.I1327V		Het	VUS	CHARGE syndrome			

**Table 5:** List of genes related to other neurological disorders.

Gene	OMIM	Sequence variant	Amino acid variant	Interpretation	Het/hom	ACMG classification	Related diseases	Co-occurring neurodevelopmental issues
CDH15	114,019	c.2024C > T	p.P675L	Nonsynonymous SNV	Het	VUS	Mental retardation	8y, development delay, behavioral disorders

GATAD2B	614,998	c.1021G > A	p.A341T	Nonsynonymous SNV	Het	VUS	Mental retardation	6y, poor language expression logic

SEMA3E	608,166	c.382A > T	p.T128S	Nonsynonymous SNV	Het	VUS	CHARGE syndrome	6y, poor language expression logic

PDE4D	600,129	c.1029A > C	p.E343D	Nonsynonymous SNV	Het	VUS	Acrodysostosis 2, with or without hormone resistance	4y, development delay

FRMPD4	300,838	c.1636C > T	p.L546F	Nonsynonymous SNV	Het	VUS	Mental retardation, X-linked	3y, speech delay, development delay, social impairment

SHROOM4	300,579	c.436C > T	p.R146W	Nonsynonymous SNV	Het	VUS	Stocco dos Santos X-linked mental retardation syndrome	3y, speech delay

ASTN2	612,856	c.1817G > A	p.R606Q	Nonsynonymous SNV	Het	VUS	Schizophrenia	3y, speech delay

DLG3	300,189	c.128G > T	p.G43V	Nonsynonymous SNV	Het	VUS	Mental retardation, X-linked	4y, speech delay, congenital heart disease, transitional endocardial cushion defect

STAG1	604,358	c.3326_3334del	p.1109_1112 del	Nonframeshift deletion	Het	VUS	Mental retardation	4y, speech delay

ASXL3	615,115	c.4004C > G	p.S1335C	Nonsynonymous SNV	Het	VUS	Bainbridge–Ropers syndrome	2y, speech delay, developmental delay

EBF3	607,407	c.1205C > T	p.A402V	Nonsynonymous SNV	Het	VUS	Hypotonia, ataxia, and delayed development syndrome	2y, speech delay, developmental delay

PER3	603,427	c.2979A > G	p.G993G	Synonymous SNV	Het	VUS	Advanced sleep phase syndrome, familial, 3	2y, speech delay, developmental delay

ERE	605,226	c.3567G > C	p.E1189D	Nonsynonymous SNV	Het	VUS	Neurodevelopmental disorder with or without anomalies of the brain, eye, or heart	4y, speech delay

NIPBL	608,667	c.5249A > G	p.Y1750C	Nonsynonymous SNV	Het	VUS	Cornelia de Lange syndrome 1	2y, speech delay, developmental delay

GRIN2A	138,253	c.3619T > C	p.Y1207H	Nonsynonymous SNV	Het	VUS	Epilepsy, with speech disorder and with or without mental retardation	3y, mental retardation, motor developmental delay and incoordination
		c.3499A > G	p.M1167V		Het	VUS		

## Data Availability

The data that support the findings of this study are available from the corresponding author upon reasonable request.

## Supporting Information

Table S1: CNVs identified through WES. Table S1 shows that all 27 CNVs identified through WES were classified as benign or of uncertain clinical significance.
