# Supplementary material for: Unveiling Hidden Genetic Architectures: Molecular Diagnostic Yield of Whole Exome Sequencing in 50 Children With Autism Spectrum Disorder Negative for Copy Number Variations
Source: Genet Res (Camb). 2025 Jul 3;2025:5724454. doi: 10.1155/genr/5724454 (PMC12245513; doi:10.1155/genr/5724454)
Supplement: Supporting Information — Additional supporting information can be found online in the Supporting Information section. [file 5724454.f1.docx]

| **Number** | **Chromosome position** | | **type of variation** | **size of  imbalance region** | **Variant classification** |
| --- | --- | --- | --- | --- | --- |
| 1 | chr1:189320000_189540000 | 1q31.1 | Del | 0.22Mb | B |
| 2 | chr1:190980001_191260000 | 1q31.2 | Dup | 0.28Mb | B |
| 3 | chr2:52200001_52440000 | 2p16.3 | Del | 0.24Mb | B |
| 4 | chr2:44000001_44400000 | 2p21 | Dup | 0.4Mb | B |
| 5 | chr2:136480001_136820000 | 2q21.3q22.1 | Dup | 0.34Mb | VUS |
| 6 | chr3:880001_1440000 | 3p26.3 | Dup | 0.56Mb | B |
| 7 | chr4:161860000_161960000 | 4q32.2 | Dup | 0.10Mb | B |
| 8 | chr4:65400000_65580000 | 4q13.1 | Dup | 0.18Mb | B |
| 9 | chr5:101400000_101700000 | 5q21.1 | Dup | 0.3Mb | B |
| 10 | chr6:95100001_95280000 | 6q16.1 | Del | 0.18Mb | B |
| 11 | chr8:2140001_2400000 | 8p23.3p23.2 | Dup | 0.26Mb | B |
| 12 | chr10:1100001_1220000 | 10p15.3 | Dup | 0.12Mb | B |
| 13 | chr10:1440001_1940000 | 10p15.3 | Dup | 0.5Mb | B |
| 14 | chr11:5480001_5920000 | 11p15.4 | Dup | 0.44Mb | B |
| 15 | chr11:97440001_97640000 | 11q22.1 | Del | 0.2Mb | VUS |
| 16 | chr12:34160001_34860000 | 12p11.1 | Dup | 0.7Mb | B |
| 17 | chr13:74860001_75200000 | 13q22.1 | Dup | 0.34Mb | B |
| 18 | chr14:22520001_22940000 | 14q11.2 | Del | 0.42Mb | B |
| 19 | chr14:106560001_106940000 | 14q32.33 | Del | 0.38Mb | B |
| 20 | chr15:32060000_32520000 | 15q13.3 | Dup | 0.46Mb | B |
| 21 | chr16:79920001_80440000 | 16q23.2 | Dup | 0.42Mb | B |
| 22 | chr18:62380001_62620000d | 18q22.1 | Dup | 0.24Mb | B |
| 23 | chr19:27740001_28660000 | 19q11q12 | Dup | 0.92Mb | B |
| 24 | chr19:56220001_56500000 | 19q13.42q13.43 | Dup | 0.28Mb | VUS |
| 25 | chrX:143280001_143520000 | Xq27.3 | Del | 0.24Mb | B |
| 26 | chrX:57760001_57900000 | Xp11.21 | Dup | 0.14Mb | B |
| 27 | chrX:132760001_133460000 | Xq26.2 | Dup | 0.7Mb | VUS |

Table S1 CNVs identified through WES

B:benign; VUS:Variant of uncertain significance
